# SARS-CoV-2 Period Seroprevalence and Related Factors, Hillsborough County, Florida, USA, October 2020–March 2021

**DOI:** 10.3201/eid2803.211495

**Published:** 2022-03

**Authors:** Anna R. Giuliano, Shari Pilon-Thomas, Michael J. Schell, Martha Abrahamsen, Jessica Y. Islam, Kimberly Isaacs-Soriano, Kayoko Kennedy, Christopher W. Dukes, Junmin Whiting, Julie Rathwell, Jonathan A. Hensel, Leslie N. Mangual, Ernst Schonbrunn, Melissa Bikowitz, Dylan Grassie, Yan Yang

**Affiliations:** Moffitt Cancer Center, Tampa, Florida, USA (A.R. Giuliano, S. Pilon-Thomas, M.J. Schell, M. Abrahamsen, J.Y. Islam, K. Isaacs-Soriano, K. Kennedy, C.W. Dukes, J. Whiting, J. Rathwell, J.A. Hensel, L.N. Mangual, E. Schonbrunn, D. Grassie, Y. Yang);; University of South Florida Morsani College of Medicine, Tampa (M. Bikowitz)

**Keywords:** COVID-19, coronavirus disease, SARS-CoV-2, severe acute respiratory syndrome coronavirus 2, viruses, respiratory infections, zoonoses, vaccine-preventable diseases, serosurvey, seroprevalence, seroepidemiologic studies, Hillsborough County, Florida, United States

## Abstract

Estimating the actual extent of the severe acute respiratory syndrome coronavirus 2 (SARS-CoV-2) pandemic is challenging because virus test positivity data undercount the actual number and proportion of persons infected. SARS-CoV-2 seroprevalence is a marker of past SARS-CoV-2 infection regardless of presence or severity of symptoms and therefore is a robust biomarker of infection period prevalence. We estimated SARS-CoV-2 seroprevalence among residents of Hillsborough County, Florida, USA, to determine factors independently associated with SARS-CoV-2 antibody status overall and among asymptomatic antibody-positive persons. Among 867 participants, SARS-CoV-2 period prevalence (October 2020–March 2021) was 19.5% (asymptomatic seroprevalence was 8%). Seroprevalence was 2-fold higher than reported SARS-CoV-2 virus test positivity. Factors related to social distancing (e.g., essential worker status, not practicing social distancing, contact with a virus-positive person, and length of contact exposure time) were consistently associated with seroprevalence but did not differ by time since suspected or known infection (<6 months vs. >6 months).

In late 2019, severe acute respiratory syndrome coronavirus 2 (SARS-CoV-2) emerged in China, ultimately leading to a global pandemic (*1*). Since January 2020, the United States has observed a dramatic rise in the incidence of SARS-CoV-2 infection, for which no endogenous immunity exists (*2*), leading to >70.6 million cases of SARS-CoV-2 and ≈860,000 deaths in the United States (*3*). Although these data provide an estimate of the infection burden, challenges exist in estimating the actual extent of the pandemic. US public health data record the number of residents that test positive for SARS-CoV-2 RNA, rates of hospitalizations, and deaths from coronavirus disease (COVID-19) among those who undergo viral testing. Missing is the proportion of the population that was ever positive for SARS-CoV-2, including those who were symptomatic but did not undergo testing and those with no or mild symptoms, where the person did not recognize COVID-19 symptoms and therefore did not undergo testing (*4*–*6*). Complicating the estimate of SARS-CoV-2 prevalence is the fact that early in the pandemic in the United States, the availability of test reagents varied on any given day at any location and recommendations for testing eligibility changed. Test positivity data likely undercounted the actual number and proportion of persons who were infected with SARS-CoV-2 (*7*,*8*). As such, the period prevalence of SARS-CoV-2 remains unknown for most communities.

Antibodies to SARS-CoV-2 begin to be detected 7 days after symptom onset (*9*) and IgG antibodies are detectable within 2 weeks after onset of infection (*10*). SARS-CoV-2 seroprevalence is a marker of past SARS-CoV-2 infection regardless of presence or severity of symptoms and therefore is a robust biomarker of infection period prevalence.

As of June 1, 2021, Florida had the third-highest number of confirmed SARS-CoV-2 cases in the United States, 2,283,315 cases (10.6% of residents), resulting in 95,210 hospitalizations and 36,869 deaths (*11*). Hillsborough County (≈1.47 million residents), where the city of Tampa is located, is one of the most populous counties in Florida. As of June 1, 2021, a total of 142,013 test-confirmed SARS-CoV-2 cases had occurred among Hillsborough County residents (9.7% of the population). The goals of this study were to estimate SARS-CoV-2 seroprevalence among Hillsborough County residents and to determine the demographic and behavioral factors independently associated with SARS-CoV-2 antibody status overall and among asymptomatic antibody-positive persons.

## Study Design

We conducted a cross-sectional study of adults residing in Hillsborough County during October 2020–March 2021. The study was approved by the Advarra Institutional Review Board and Moffitt Cancer Center’s Scientific Review Committee. The University of Florida’s Bureau of Economic and Business Research drew the study population from the greater Hillsborough County by using randomly selected mailing addresses. Adults >18 years of age who were free of fever at the time of interview were eligible for the study. To ensure an adequate sample size of residents across the lifespan, we aimed to enroll relatively equal numbers of persons (balanced on sex) in each of 4 age groups: 18–34, 35–54, 55–64, and >65 years. We contacted potential participants by mail and email to inform them of the study. If they agreed to participate, they were scheduled for an in-person blood draw after completion of a web-based eligibility criteria checklist and informed consent form and a short questionnaire that captured demographic information, SARS-CoV-2 exposure history, underlying conditions, immunosuppression status, and use of immunosuppressive medications. Participants received a $25 gift card after completing the blood draw.

### Selection of Hillsborough County Residents for Study

The Bureau of Economic and Business Research created a random representative sample of Hillsborough County residents by using the address-based sample method, a probability-based frame of street addresses that relies on the US Postal Service Computerized Delivery Sequence File. Because this file contains >147 million residential addresses, the address-based sample frame covers nearly 100% of all households in the country. We sent letters and postcards to potential participants inviting them to go to the study website and complete a brief form to indicate their interest in participating. In addition, emails were sent to a randomly selected population by eTargetMedia (https://www.etargetmedia.com), a multichannel marketing company with a detailed database of email addresses. 

The study webpage described the study rationale and assessed eligibility criteria, which included residency in Hillsborough County and age of >18 years. At the time of the scheduled clinic visit, eligibility for the blood draw also included not currently experiencing COVID-19 symptoms (e.g., being free of fever [body temperature <100.4°F, as assessed using a noncontact infrared thermometer], cough, and shortness of breath). Eligible potential participants were then directed to an online informed consent form to review and sign and a brief questionnaire to complete before scheduling a date and time for the blood draw.

Forty thousand letters or postcards and 10,000 emails were sent to Hillsborough County addresses. A total of 1,621 residents completed the eligibility questionnaire, and 1,571 were eligible to participate. Of those eligible, 1,135 electronically signed a consent form, 1,038 completed the online questionnaire, and 922 completed the study visit and blood draw. Fifty-five of the study participants had received >1 COVID-19 vaccine doses and were excluded from the analyses, resulting in a final sample size of 867.

## Study Procedures

### Data Acquisition and Management

Persons who were contacted through postal mail were provided a link to a website that enabled authentication using a unique identifier they were assigned. After authenticating, persons were shown a webpage with a brief description of the study. Those who chose to proceed were asked to electronically sign the informed consent form. Participants were then asked to complete a short questionnaire and contact the research clinic to schedule an appointment for a blood draw. If participants did not contact the research clinic within 3 days of completing the online questionnaire, the research staff contacted the person to schedule an appointment. The questionnaire collected information related to sociodemographic information, SARS-CoV-2 exposure history (self-reported in exposure hours per day), past COVID-19 symptoms, underlying conditions associated with increased infection and disease risk, immunosuppression status, and use of immunosuppressive medications.

### Clinic Procedures

After we verifying participants’ identities and their completion of the required forms, participants attended an in-person clinic visit at Moffitt Cancer Center’s Research Clinic. All staff and study participants were required to wear facemasks at all times, no-touch temperature screening was used, and questions regarding respiratory illness were asked. One tiger top tube of blood was drawn per participant. We processed blood by letting it stand for 20–60 min to clot and then spun it for 20 min at 3,200 rpm, and placed it in a refrigerator until couriered to the laboratory. We aliquoted and then maintained serum samples at –80°C until antibody analysis. Before antibody testing, we heat-inactivated all serum samples in a 56°C water bath for 1 h.

### SARS-CoV-2 IgG Antibody Assay

To evaluate serostatus, we performed a 2-step ELISA adapted from the Krammer (Icahn School of Medicine at Mount Sinai) protocol, which measured IgG responses against the receptor-binding domain and spike protein (*12*,*13*). In brief, a high-throughput screening of samples against receptor-binding domain was followed by a second step in which positive samples underwent a confirmatory ELISA against the full-length spike protein. We diluted presumptive positives 1:100, 1:300, 1:900, 1:2,700, and 1:8,100, and used goat anti–human ​​​horseradish peroxidase–conjugated antibody (diluted 1:5,000) as the secondary antibody. We designated as positive the samples having 2 consecutive dilutions with optical density values >3 × SD of the mean of the negative controls. Negative controls included serum pools collected before 2020. Positive controls included convalescent serum from SARS-CoV-2–positive patients or monoclonal antibodies against SARS-CoV-2 proteins (L. Pinto, Frederick Laboratories, National Institutes of Health, pers. comm., emails, April and October 2020). Assay sensitivity was 96.8%, and specificity was 94.1% (Appendix).

### Data Analyses

We summarized sociodemographic and behavioral characteristics by using descriptive statistics. We compared SARS-CoV-2 antibody positivity across participant sociodemographic and behavioral characteristics by using bivariate analyses, specifically Fisher exact test or χ^2^ test as appropriate. We evaluated associations between SARS-CoV-2 antibody positivity with potential predictors by using univariate logistic regression analyses. We developed the fully adjusted model by using a backward elimination approach; specifically, we removed variables with p values >0.25 from the final model. We included the following variables in the backward selection model: age, sex, race, ethnicity, marital status, smoking status, living with chronic disease, lung problem, work environment during pandemic, practiced mask use since start of pandemic, practiced social distancing since start of pandemic, mean hours/week interacting with virus-positive contact, traveled out of state after February 2020, relationship to virus-positive contact, avoid groups of people, only going outside the home for essential trips, and ever had COVID-19 symptoms. We performed all analyses by using SAS 9.4 (https://www.sas.com) and RStudio 4.0.2 (https://www.rstudio.com).

## Results

Among 867 COVID vaccine-naive Hillsborough County residents, 19.5% (95% CI 16.9%–22.3%) tested antibody-positive (Appendix Table); adjusted prevalence of 15% did not differ significantly from the crude estimate. The median age of study participants was 50 years (interquartile range 38–61 years), and 65.7% were women. Most participants were White (82.7%), non-Hispanic (83.2%), never smokers (74.2%), and immunocompetent (91.8%). Eighteen percent reported essential worker status (i.e., employed in either a hospital, clinic, grocery store, or in a public services industry). Approximately 60% had either never been exposed to a SARS-CoV-2–positive person or were not sure if they had been exposed. Nearly all respondents (99.5%) indicated they had changed their behavior since the pandemic started (data not shown); 96.1% reported wearing a mask outside of the home fairly often or often, and 89% reported keeping >6 feet away from other persons since the start of the pandemic. Approximately 30% (30.2%) reported ever having COVID-19 symptoms, although 14.5% reported testing virus positive. Only 7 participants had been hospitalized because of COVID-19.

We observed no differences in SARS-CoV-2 seroprevalence by sex, age group, race, or ethnicity (Appendix Table). Seroprevalence was higher among those living with lung disease (27.2%), especially persons who reported having asthma (28.1%), and lower among those living with an autoimmune disease (9.9%). Reported exposure to virus-positive persons was significantly associated with higher seroprevalence; 35.8% of those reporting contact with a documented positive person and 36.7% reporting contact with a presumed positive person were antibody-positive. In addition, 44.9% seroprevalence occurred among those whose virus-positive contact was a family member. More than 96% of those who tested positive for SARS-CoV-2 RNA (97% for those infected <6 months ago and 96.2% if positive >6 months ago) were antibody-positive. Forty-five percent of those who reported having COVID-19 symptoms were antibody-positive, and 8.3% of those who reported never having COVID-19 symptoms were antibody-positive, which we refer to as asymptomatic infection. Seroprevalence did not significantly differ by reported social-distancing or mask-wearing practices. We noted the relationship between hours per week exposed to a virus-positive person and antibody-positivity (Figure). The percentage testing antibody-positive increased with increasing exposure time but plateaued at ≈50% seroprevalence at >48 hours of exposure per week.

**Figure:**
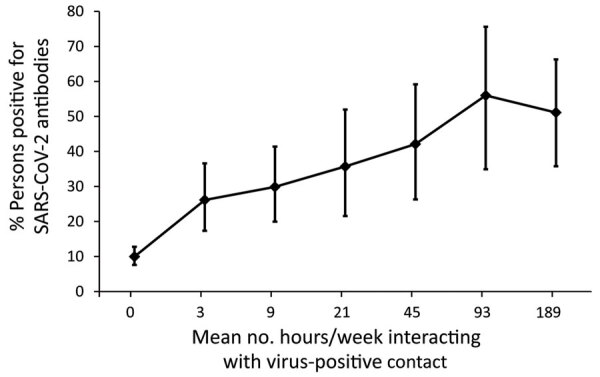
Relationship between mean number of hours per week exposed to a SARS-CoV-2–positive person and antibody positivity among residents, Hillsborough County, Florida, USA, October 2020–March 2021. Error bars indicate 95% CIs. SARS-CoV-2, severe acute respiratory syndrome coronavirus 2.

We noted factors independently associated with testing positive for SARS-CoV-2 antibodies (Table 1). Potential exposure to a virus-infected person increased the odds of testing antibody-positive, including essential workers employed in a hospital, clinic, grocery store or other public services industry (adjusted odds ratio [aOR] 2.40 [95% CI 1.42–4.07]), contact with a virus-positive family member (aOR 4.62 [95% CI 2.49–8.58]) or friend (aOR 4.22 [95% CI 2.44–7.30]), not avoiding groups of people (aOR 1.71 [95% CI 1.06–2.76]), and mean hours per week exposed to the virus-positive person (adjusted continuous odds ratio 1.01 [95% CI 1.00–1.01]). Odds of testing antibody-positive were high among those who reported ever experiencing COVID-19 symptoms (aOR 9.14 [95% CI 5.93–14.08]). We observed significantly lower odds of testing antibody-positive among divorced, separated, or widowed persons (aOR 0.40 [95% CI 0.20–0.77]) and those living with a chronic illness (aOR 0.56 [95% CI 0.34–0.93]).

**Table 1:** Factors associated with severe acute respiratory syndrome coronavirus 2 antibody prevalence among residents, univariate and multivariable models, Hillsborough County, Florida, USA, October 2020–March 2021*

Characteristic	Unadjusted OR (95% CI)	Adjusted OR† (95% CI)
Age group, y		
18–44	Referent	Referent
45–54	0.88 (0.56–1.37)	1.13 (0.64–1.98)
55–64	0.71 (0.46–1.12)	0.86 (0.48–1.55)
>65	0.71 (0.43–1.18)	1.64 (0.80–3.35)
Sex		
M	Referent	Referent
F	0.95 (0.67–1.35)	0.88 (0.57–1.38)
Marital status		
Married or living together	Referent	Referent
Single, never married	0.91 (0.60–1.37)	1.05 (0.61–1.80)
Divorced, separated , or widowed	0.49 (0.28–0.86)	0.40 (0.20–0.77)
Living with chronic disease‡	0.88 (0.61–1.28)	0.56 (0.34–0.93)
Essential worker status		
Not essential worker	Referent	Referent
Hospital, clinic, grocery store, public services	1.89 (1.26–2.84)	2.40 (1.42–4.07)
Financial services, banking, or other	0.91 (0.55–1.52)	0.70 (0.37–1.31)
Mean hours/week interacting with virus-positive contact	1.02 (1.01–1.02)	1.01 (1.001–1.01)
Relationship to virus-positive contact		
No known contact with virus-positive person	Referent	Referent
Family member	8.79 (5.62–13.77)	4.62 (2.49–8.58)
Friend or other	5.52 (3.43–8.90)	4.22 (2.44–7.30)
Co-worker	2.89 (1.51–5.53)	2.04 (0.95–4.40)
Does not avoid groups of people	1.65 (1.12–2.43)	1.71 (1.06–2.76)
Ever had coronavirus disease symptoms	9.24 (6.33–13.48)	9.14 (5.93–14.08)

*Based on a 863-person sample size. The following variables were included in the backward selection modeling approach: age, sex, race, ethnicity, marital status, smoking status, living with chronic disease, lung problem, work environment during pandemic, practiced mask use since start of pandemic, practiced social distancing since start of pandemic, mean hours/week interacting with virus-positive contact, traveled out of state after February 2020, relationship to virus-positive contact, avoid groups of people, only going outside the home for essential trips, and ever had coronavirus disease symptoms. OR, odds ratio. †Results from the full model, which included all variables, listed in Appendix Table. ‡Including cancer, heart disease, diabetes, autoimmune disease, kidney disease, liver disease, and being immunosuppressed.

Among 605 participants who reported never having COVID symptoms, 50 tested antibody-positive and are referred to as having asymptomatic infection (Appendix Table). Essential worker status (aOR 2.28 [95% CI 1.13–4.60]), interacting with a virus-positive friend (aOR 3.72 [95% CI 1.71–8.11]), and not avoiding groups of people (aOR 2.90 [95% CI 1.53–5.50]) were independently associated with having asymptomatic infection (Table 2).

**Table 2:** Factors associated with severe acute respiratory syndrome coronavirus 2 antibody prevalence among asymptomatic participants, univariate and multivariable models, Hillsborough County, Florida, USA, October 2020–March 2021*

Characteristic	Unadjusted OR (95% CI)	Adjusted OR (95% CI)†
Age group, y		
18–44	Referent	Referent
45–54	0.95 (0.47–1.92)	1.04 (0.49–2.21)
55–64	0.48 (0.21–1.11)	0.57 (0.23–1.38)
>65	0.43 (0.16–1.18)	0.69 (0.24–2.03)
Sex		
M	Referent	Referent
F	1.11 (0.60–2.06)	0.91 (0.47–1.78)
Essential worker status		
Not essential worker	Referent	Referent
Hospital, clinic, grocery store, public services	2.44 (1.28–4.65)	2.28 (1.13–4.60)
Financial services, banking, or other	0.76 (0.28–2.02)	0.55 (0.19–1.58)
Mean hours/week interacting with virus-positive contact	1.01 (1.01–1.02)	1.01 (1.00–1.02)
Relationship to virus-positive contact		
No known contact with virus-positive person	Referent	Referent
Family member	4.23 (1.95–9.16)	2.67 (0.94–7.59)
Co-worker	2.32 (0.83 −6.52)	1.89 (0.62–5.73)
Friend or other	4.40 (2.10–9.21)	3.72 (1.71–8.11)
Does not avoid groups of people	3.03 (1.66–5.56)	2.90 (1.53–5.50)

*Based on a 602-person sample size. The following variables were included in the backward selection modeling approach: age, sex, race, ethnicity, marital status, smoking status, living with chronic disease, lung problem, work environment during pandemic, practiced mask use since start of pandemic, practiced social distancing since start of pandemic, mean hours/week interacting with virus-positive contact, traveled out of state after February 2020, relationship to virus-positive contact, avoid groups of people, only going outside the home for essential trips, and ever had coronavirus disease symptoms. OR, odds ratio. †Results from the full model, which included all variables, listed in Appendix Table.

## Discussion

Overall, ≈20% of study participants in this single Florida county had evidence of past infection with SARS-CoV-2, approximately 2-fold higher than the period prevalence of confirmed SARS-CoV-2 infections reported by the Florida Department of Health (10.6% through June 1, 2021) (*11*). This finding is not surprising given that molecular testing was not widely available early in the pandemic and, when it was available, not all persons with symptoms sought testing, and some never experienced symptoms; thus, many who were infected were undercounted in public health databases. A key finding of this study is that nearly 100% of persons who had confirmed or suspected infection were antibody-positive and remained antibody-positive even if the infection occurred >6 months before antibody testing. A question that remains unanswered by our analysis and other studies is the duration of the antibody response among those who experienced infection with SARS-CoV-2.

The seroprevalence estimated in our study demonstrates that, by March 2021, 1 in 5 adults residing in Hillsborough County may have been previously infected with SARS-CoV-2. This seroprevalence is higher than that found in other studies conducted during a similar timeframe of the COVID-19 pandemic; however, differences in SARS-CoV-2 seroprevalence are highly influenced by geographic location and the populations included in the study. For example, a seroprevalence study conducted in Virginia during June–August 2020 found that, although the overall prevalence estimated was low at 2.4%, the range by ZIP code varied from 0% to 20% (*7*). Similar to our study, the seroprevalence of 2.4% in Virginia was 2.8 times higher than the confirmed case counts. This ratio is relatively low compared with previous studies conducted in the United States, which have shown 6–53 times more infections than those ascertained by confirmed case counts (*8*,*14*,*15*). Differences in underascertainment across studies evaluating seroprevalence may be attributed to differences in the population included, timing of the epidemic across regions, and differences in test characteristics of assays used.

A nationwide study conducted by the Centers for Disease Control and Prevention that examined residual clinical samples from inpatients and outpatients found seroprevalence ranged from 0% in South Dakota (August 10–27, 2020) to 23% in New York State (July 27–August 13, 2020) (*16*). This analysis, which used commercial assays, found a seroprevalence of 8.5% for the state of Florida in September 2020. A prior Florida seroprevalence study of >5,500 healthcare workers and first responders tested in early summer of 2020 observed a seroprevalence of 4.1% (range 2.6%–8.7%) (*17*). However, considerable heterogeneity in SARS-CoV-2 antibody prevalence among first responders and healthcare workers was observed; those residing in Miami–Dade County and adult members of racial and ethnic minority populations, including Haitian, Creole, non-Hispanic Black, and Hispanic or Latino, were more likely to be seropositive (*17*). Similarly, a nationwide study of adults who had never had COVID-19 diagnosed found a seroprevalence of 4.6%; higher prevalence was found among adults living in early outbreak locations, Black adults, Hispanic adults, and adults residing in urban areas (*18*). Although our study did not demonstrate racial disparities, we observed that essential workers, including those working in grocery stores, had higher odds of SARS-CoV-2 antibody positivity. Because persons in minority communities are more likely to hold such occupations (*19*), our results contribute to the literature demonstrating the disproportionate burden of COVID-19 in the United States among vulnerable populations such as racial and ethnic minorities.

In our study, mask wearing was not associated with antibody status. This lack of an association is likely attributable to several factors. Nearly 100% of respondents reported mask usage, so social desirability likely influenced responses to this question. In addition, we did not assess detailed information regarding mask use, such as type of mask, situations in which mask wearing occurred, and consistency of proper mask usage, so our study cannot adequately assess the protection conferred by mask usage. In contrast, social-distancing behaviors consistently emerged as a factor associated with risk for infection, despite 11% of participants reporting that they never, rarely, or almost never practiced social distancing. This behavior included not avoiding crowds and interacting with a known or suspected virus-positive family member, co-worker, or friend. The percentage seropositive increased with increasing hours per week exposed to an infected person. We did not ask participants to report the physical distance they maintained, so we cannot assess what distance in feet was associated with protection.

Throughout the pandemic, much discussion has occurred about the dangers of infection for those with autoimmune disease and their greater susceptibility to illness. Study participants with autoimmune disease or on immunosuppressant medication had lower rates of antibody positivity, roughly half that of the overall study population. This finding is likely attributable to extra precautions taken to avoid infection as opposed to a reflection of actual susceptibility to illness.

Despite some study participants reporting a large number of hours per week exposed to a person with COVID-19 symptoms, only ≈50% became infected themselves. Infection rates among adults with high exposure to COVID-19 is likely multifactorial and varies from population to population on the basis of contextual factors, which can be demonstrated by seroprevalence studies conducted in large healthcare systems of healthcare workers with high exposure to COVID-19. For example, the seroprevalence of healthcare personnel tested during June–August 2020 throughout all Mayo Clinic facilities was only 0.6%, and areas undergoing greater community disease transmission and burden were associated with higher seroprevalence among healthcare providers (*20*). Notably, the Mayo Clinic in Florida had a seroprevalence of 0.8%. In contrast, the seroprevalence of healthcare workers in New York City during the same period was 13.7% (*21*). The marked difference in seroprevalence among these highly exposed adults may be caused by differences in hotspots or outbreaks of SARS-CoV-2 infection in the regions each health system is located, as well as differences in access to personal protective equipment and adherence to precautions including wearing masks. Similarly, differences we observed in our study may be caused by variability in adherence to preventive behaviors, such as prompt isolation from persons with COVID-19, or the household setting and environment. We observed that the odds of SARS-CoV-2 seroprevalence was highest among those with a family member as a known virus-positive contact. In shared family spaces where social distancing may not be possible, risk for household transmission is high (*22*). Asymptomatic transmission before the onset of symptoms in a household is also highly probable.

A low percentage of antibody-positive persons never had COVID-19 symptoms, what we refer to as the asymptomatic infection prevalence. Surprisingly, this percentage was relatively low (≈8%) and was not associated with age. The only factors significantly associated with asymptomatic infection were those related to social distancing, whether that was not avoiding crowds, contact with a friend who was virus-positive, or repeated contact with community members as an essential worker.

A strength of this study is the relatively large sample size and inclusion of a broad range of ages. We captured participant information regarding factors that may be associated with susceptibility to infection, protective behaviors practiced, and exposure and length of exposure to SARS-COV-2–infected persons. Although invitations were sent at random to county residents, a small rate of participation resulted from this recruitment method. Some residents may have received multiple invitations (i.e., email and letter, or email and postcard). Because of the low rate of participation, we were not able to obtain a representative sample of the underlying county population. However, the study enrolled persons from 53 (96%) of the 55 ZIP codes associated with Hillsborough County. The final study sample included a higher proportion of women than men, and participants were predominantly non-Hispanic White. Many reported exposure to an infected person, so the seroprevalence we report may be an overestimate of the actual period prevalence. Hillsborough County includes the city of Tampa as well as both rural and suburban communities. Although the area is relatively densely populated, few residents use public transportation; this county is less densely populated than other urban counties in Florida, such as Miami–Dade County. Therefore, the period seroprevalence we have reported may be lower than what would be observed in city centers.

The estimates of seroprevalence from our study demonstrate that the cumulative case numbers confirmed through molecular RNA–based testing likely underrepresent the actual number of cases of SARS-CoV-2 infection in the United States and Florida. Frequency of contact with family or friends with confirmed COVID-19 diagnoses was strongly associated with being SARS-CoV-2 antibody–positive, indicating the importance of social distancing, particularly from friends or family with confirmed COVID-19. The availability of vaccination should help alleviate disparities in SARS-CoV-2 positivity observed for higher risk groups because of structural and occupational factors, such as among essential workers and those with frequent contact with persons with confirmed COVID-19. This analysis should inform the broader ongoing policy in the United States regarding the relative benefits of recommended mitigation strategies against the spread of SARS-CoV-2.

Appendix Additional information about SARS-CoV-2 period seroprevalence and related factors, Hillsborough County, Florida, USA, October 2020–March 2021.
