# Supplementary material for: SARS-CoV-2 Period Seroprevalence and Related Factors, Hillsborough County, Florida, USA, October 2020–March 2021
Source: Emerg Infect Dis. 2022 Mar;28(3):556–63. doi: 10.3201/eid2803.211495 (PMC8888241; doi:10.3201/eid2803.211495)
Supplement: Appendix — Additional information about SARS-CoV-2 period seroprevalence and related factors, Hillsborough County, Florida, USA, October 2020–March 2021. [file 21-1495-Techapp-s1.pdf]

Article DOI: https://doi.org/10.3201/eid2803.211495

SARS-CoV-2 Period Seroprevalence and Related Factors, Hillsborough County, Florida, USA, October 2020–March 2021

Appendix

Protein Expression and Purification

Expression plasmids for full-length spike protein (BEI resources, NR-52394) and receptor-binding domain (RBD) (BEI resources, NR-52309) in pCAGGS vectors with C-terminal His_6 affinity tags were provided by the Krammer laboratory. Expi293F cells (ThermoFisher, A14528) were maintained at 0.5–3 million cells/mL, 37°C, 8% CO_2, 140 rpm (*1*). Cells at a density of 3 million cells/mL were split to 1 million cells/mL 24 hours before transfection. The transfection reagent consisted of sterile PBS (66 µL/mL culture), DNA (1 µg/mL culture) and Transporter 5 (PolySciences, 26008) (4 µL/mL culture). Transfected cells were grown at 33°C (120 rpm) for 72 hours (>90% cell viability), and the supernatant was harvested in two centrifugation steps (400xg for 20 min and 4,000xg for 20 min), filtered through 0.45 um low protein binding filter, and concentrated at 4°C via a Hydrosart cross flow cassette (Sartorius, VF05H4) using a 100 kDa filter (Vivaflow) to ≈500 mL.

Five M filtered NaCl was slowly added to the concentrated supernatant until 300 mM NaCl was achieved and loaded at 0.5 mL/min onto a 58 mL Ni Sepharose Excel (Cytiva, 17371202) FPLC column at 4°C equilibrated with 50 mM Na/K phosphate buffer (pH 7.4) and 500 mM NaCl. A gradient from 0 – 500 mM imidazole over 5 column volumes was applied to elute the target protein. Fractions containing the target protein were combined and purified to homogeneity by size exclusion chromatography using HiLoad Superose 6 16/600 (Fisher Scientific, 29323952) in PBS. Spike or RBD-containing fractions were combined, concentrated to ~1 mg/mL flash-frozen in liquid N_2 and stored at −80°C. The yields were 8.5 mg of RBD and 1 mg of full-length Spike per liter of culture, and the proteins were of >90% purity as judged by SDS-PAGE.

SARS-CoV-2 Antibody Assay Methods

Wells of 96-well flat-bottom plates were coated with 50µL of recombinant RBD protein at a concentration of 2 µg/mL and incubated O/N at 4°C. Wells were washed using 200 µL of PBST/well 3X and 200 µL of 3% Blocking Buffer (milk powder in PBST) was added to each well and incubated for 1 hour. Plates were washed with 1% Blocking Buffer and 100 µL of diluted serum samples (1:50) were added. After a 2-hour incubation, plates were washed as above and 50 µL of 1:5,000 diluted goat anti-human conjugated with HRP in 1% Blocking Buffer was added to each well. Plates were incubated in the dark for 1-hour at room temperature. Following 5 washes, 100 µL of SigmaFAST OPD Solution (Sigma-Aldrich) was added per the manufacturer's suggested protocol. After a 10-minute incubation in the dark, 50 µL of 5N H_2SO_4 was added to each well. Plates were read on a plate reader at an OD of 490nm. Samples that exceeded 3X the standard deviation of the mean of the negative controls were designated presumptive positives. The SPIKE ELISA was performed as above with the following changes: 50 µL of recombinant SPIKE protein was plated at a concentration of 2 µg/mL.

Reference

1. Stadlbauer D, Amanat F, Chromikova V, Jiang K, Strohmeier S, Arunkumar GA, et al. SARS-CoV-2 seroconversion in humans: a detailed protocol for a serological assay, antigen production, and test setup. Curr Protoc Microbiol. 2020;57:e100. PubMed https://doi.org/10.1002/cpmc.100

Appendix Table. SARS-CoV-2 antibody prevalence among residents, by sociodemographic, susceptibility, and behavioral characteristics, Hillsborough County, Florida, USA, October 2020–March 2021*

Characteristic	Overall, no. (%)	Antibody positive, no. (%)	p-value
Total	867 (100)	169 (19.5 [16.9–22.3])†	
Sex			
F	570 (65.7)	110 (19.3)	0.77
M	293 (33.8)	59 (20.1)	
Age group, y‡			
18–44	316 (36.4)	70 (22.2)	0.40
45–54	190 (21.9)	38 (20.0)	
55–64	213 (24.6)	36 (16.9)	
≥65	148 (17.1)	25 (16.9)	
Race			
White	717 (82.7)	139 (19.4)	0.85
Black or African American	44 (5.1)	8 (18.2)	
Asian	51 (5.9)	9 (17.6)	
Other	55 (6.3)	13 (23.6)	
Ethnicity			
Hispanic or Latin	146 (16.8)	35 (24.0)	0.14
Not Hispanic or Latin	721 (83.2)	134 (18.6)	
Marital status			
Single, never married	192 (22.1)	38 (19.8)	0.04
Married or living together	539 (62.2)	115 (21.3)	

Characteristic	Overall, no. (%)	Antibody positive, no. (%)	p-value
Divorced, separated, or widowed	136 (15.7)	16 (11.8)	
Education			
No college	57 (6.6)	8 (14.0)	0.22
Some college	160 (18.5)	30 (18.8)	
Graduated college	383 (44.2)	86 (22.5)	
Postgraduate or professional school	267 (30.8)	45 (16.9)	
Smoking status			
Never	643 (74.2)	124 (19.3)	0.79
Former or current	224 (25.8)	45 (20.1)	
Underlying condition			
Cancer	16 (1.8)	3 (18.8)	1.00
Heart disease	159 (18.3)	30 (18.9)	0.91
Diabetes	55 (6.3)	15 (27.3)	0.16
Lung disease	103 (11.9)	28 (27.2)	0.05
Type of lung disease			
Asthma	89 (10.3)	25 (28.1)	0.08
Chronic obstructive pulmonary disease	14 (1.6)	3 (21.4)	
Autoimmune disease	71 (8.2)	7 (9.9)	0.04
Kidney or liver disease	19 (2.2)	4 (21.1)	0.78
On immunosuppressant medications	18 (2.1)	2 (11.1)	0.55
SARS-CoV-2 exposure risk			
Traveled out of country after February 2020	65 (7.5)	14 (21.5)	0.67
Traveled out of state after February 2020	447 (51.6)	96 (21.5)	0.13
Work environment during pandemic			
Work from home	196 (22.6)	35 (17.9)	0.80
Work away from home	170 (19.6)	37 (21.8)	
Work an alternating schedule	113 (13.0)	23 (20.4)	
No work, student, or retired	388 (44.8)	74 (19.1)	
Essential worker status			
Hospital, clinic, grocery store, public services	156 (18.0)	45 (28.8)	0.01
Financial services, banking, or other	134 (15.5)	22 (16.4)	
Not essential worker	577 (66.6)	102 (17.7)	
Ever interacted with documented or presumed virus-infected person			
No	174 (20.1)	13 (7.5)	<0.001
Don't know	345 (39.8)	31 (9.0)	
Yes, documented virus-positive	299 (34.5	107 (35.8)	
Yes, presumed virus-positive	49 (5.7)	18 (36.7)	
Relationship to virus-positive contact			
Family member	147 (17.0)	66 (44.9)	<0.001
Co-worker	71 (8.2)	15 (21.1)	
Friend or other	130 (15.0)	44 (33.8)	
Behavior since pandemic started			
Practiced mask use since start of pandemic			
Never, almost never, or sometimes	34 (3.9)	5 (14.7)	0.82
Fairly often	104 (12.0)	21 (20.2)	
Often	729 (84.1)	143 (19.6)	
Practiced social distancing since start of pandemic			
Never, almost never, or sometimes	95 (11.0)	21 (22.1)	0.50
Fairly often	257 (29.6)	54 (21.0)	
Often	515 (59.4)	94 (18.3)	
Tested virus positive any time since February 2020			
Never	348 (40.1)	24 (6.9)	<0.001
<6 mo ago	100 (11.5)	97 (97.0)	
≥6 mo ago	26 (3.0)	25 (96.2)	
Ever had COVID-19 symptoms			
Yes	262 (30.2)	119 (45.4)	<0.001
No	605 (69.8)	50 (8.3)	
Hospitalized because of COVID-19	7 (0.8)	7 (100)	1.00

*All values are no. (%) except where indicated. COVID-19, coronavirus disease; SARS-CoV-2, severe acute respiratory syndrome coronavirus 2.
†95% CI of antibody positivity.
‡Median age 50 years (interquartile range 38–60 years).
